# Intermolecular binding between bulk water and dissolved gases in earth’s magnetic field

**DOI:** 10.1371/journal.pone.0267391

**Published:** 2022-05-12

**Authors:** Masahiro Kohno, Toshiaki Kamachi, Koji Fukui

**Affiliations:** 1 Department of Bioscience and Engineering, College of Systems Engineering and Science, Shibaura Institute of Technology, Minuma-ku, Saitama, Japan; 2 Department of Life Science and Technology, Tokyo Institute of Technology, Ookayama Meguro-ku, Tokyo, Japan; Beijing Foreign Studies University, CHINA

## Abstract

Elucidation of the static states and dynamic behavior of oxygen and nitrogen dissolved in water is one of the most important issues in the life sciences. In the present study, experimental trials and theoretical calculations were performed based on the hypothesis that the dissolution of gas molecules in water is related to excitation by the Earth’s magnetic field. Using quantum theories such as those used to describe electro magnetic resonance and nuclear magnetic resonance, this study investigated the states of oxygen, nitrogen and hydrogen dissolved in water. The results indicate that the Earth’s magnetic field is involved in the bonding and dissociation of molecules at the gas-liquid interface. These calculations assessed the effect of a field strength of 1.0 x 10^−4^ T and reproduced the influences of temperature changes on dissolved gas concentrations. Molecular interactions caused by electromagnetic properties and the external geomagnetic field were found to affect intermolar bonding associated with water cluster structures. It is concluded that the binding between molecules typically attributed to Coulomb coupling by magnetic charge and van der Waals forces results from excitation in the Earth’s magnetic field.

## Introduction

Both water and oxygen are essential to life as we know it. Water is a solvent for numerous inorganics or organic substances and plays a major role in the transport of substances in living tissues. Water is also a liquid over a wide temperature range and has a high specific heat capacity and heat of evaporation, such that it has the ability to buffer thermal changes [1–3]. As an example, water molecules readily undergo strong hydrogen bonding, leading to the formation of clusters between molecules [4]. The various physical and chemical properties of water are of interest and have been extensively studied [5,6].

Water is also able to bind with other substances in a process termed hydration, based on effects such as dipole orientation [7,8]. On the other, dissolved oxygen gas has played a vital role in the evolution of life on Earth, its ability to donate unpaired electrons is employed in metabolic energy pathways [9–12]. Other elements that are typically gases at ambient temperature are also of interest biologically [13,14]. Nitrogen is an essential element for all living organisms on the planet [15]. However, the dynamic behavior of nitrogen dissolved in water is difficult to ascertain, and the role of dissolved nitrogen in metabolic processes is not well understood at present. Hydrogen is also involved in metabolic processes and exhaled in the breath of mammals. Recently, hydrogen dissolved in water has been shown to have pharmacological properties, leading to increased interest in the biological functions of the hydrogen molecule [16–18]. And also, it has also been shown that water can be affected by the magnetic field [19–23]. In our previous papers, the application of ultrasonic waves to water containing dissolved gases such as oxygen, nitrogen or hydrogen has been found to generate various reactive species, including hydroxyl radicals (HO•) and hydrogen radicals (H•). These radicals changed depending on the type of dissolved gas and water temperature [24,25].

The purpose of the present study was to assess changes in the state of static water containing dissolved gas molecules. Theoretical analyses based on the same quantum theory used to explain nuclear magnetic resonance (NMR) and electron paramagnetic resonance (EPR) were performed [26–28]. These calculations examined using nuclear spin moment (I) of water molecules, electron spin moment (S) of oxygen, electron magnetic moment of nonpolar hydrogen molecules and of quadrupolar nitrogen molecules. In addition, changes in the excitation energy (ΔE) in the presence of an external magnetic field related to dissolved gases were determined. The results suggest that the electromagnetic properties of the magnetic moment of the proton (*μ*_p_) and the magnetic moment of the electron (μ_e_) play a role in the binding and dissociation mechanisms for water. The data indicate that the intermolecular interactions typically described as Coulomb force due to magnetic charge and van der Waals forces are in fact induced by the Earth’s geomagnetic field of 1.0 x 10^−4^ T [29].

## Material and methods

### Chemicals

Oxygen and hydrogen gas (99.5% minimum purity) were purchased from the Japan Fine Products Co. (Kawasaki, Kanagawa, Japan).

### Analytical methods

Ultrapure water (specific resistance: 18.2 MΩ cm) was used in all trials. Dissolved oxygen and nitrogen levels were determined with an oxygen electrode (model 5905/5010 BOD, YSI, Yellow Spring, OH) and hydrogen electrode (DH-35A DH-meter, TOA-DDK Corp., Tokyo, Japan). In these trials, oxygen and hydrogen were passed through ultrapure water held at a temperature in the range of 5 to 60°C until equilibrium concentrations were achieved, following which the levels of these gases were ascertained using the electrodes. The averages of duplicate trials are reported herein. The nitrogen concentrations in water employed in this work were obtained from previous publications. Electron paramagnetic resonance (EPR) analyses of dissolved oxygen were performed with an electron spin resonance spectrometer (model FA200, JEOL, Tokyo, Japan).

### Electromagnetic theory of intermolecular interactions

In electromagnetism, repulsion occurs between electrical charges of the same sign, and attraction occurs between charges of different signs. According to Coulomb theory, the strength of the interaction between molecules is proportional to the product of the charges, and is inversely proportional to the square of the distance between them. In the present study, we test the hypothesis that charge contributes to the mechanism by which gas molecules such as oxygen, nitrogen, and hydrogen dissolve in water. Gas molecules that have an electrical charge have an electromagnetic energy (Δ*E*) excited by the magnetic field of the Earth. For example, O_2_ is a paramagnetic molecule that has a magnetic moment due to its electron spin, H_2_ is a non-polar molecule that has an electrical quadrupole moment, and nitrogen also has a quadrupole moment. In contrast, water is a dipolar molecule that exhibits a nuclear magnetic moment due to protons. Oxygen has two unpaired electrons per molecule, and has a unique biradical electron configuration. Normally, free radical molecules have a single unpaired electron (free electron: e^-^). EPR theory states that the electron spin moment (*S* = ±1/2) represents the state of an unpaired electron. Furthermore, when an oxygen molecule, which has two electrons, is placed in a magnetic field, the electron spin moment becomes *S* = ±1 because precession occurs since the rotational motion is limited. Because the actual value of *S* for oxygen has not been reported, we determine it by EPR measurements of oxygen gas.

### Relationship between multipolar moment and magnetic moment

Oxygen is a bi-radical with a unique electron configuration, having two unpaired electrons in each molecule [13]. Normally, a free radical molecule has one unpaired electron (that is, a free electron: e-). According to the theory of EPR, a single unpaired electron will have an electron spin moment (S = ± 1/2). When an oxygen molecule with two electrons is placed in a magnetic field, the rotational motion will be restricted such that precession occurs, and so the electron spin moment (S) is S = ± 1. The spin angular momentum (*S*) under these conditions is defined as *S* = S (S + 1) ^1/2^. Since the spin moment (S) of oxygen has not yet been reported, this value was determined in the present work based on the EPR analysis of gaseous oxygen.

H_2_O is a dipolar molecule in which the atomic nuclei are polarized due to the molecular structure, and nuclear spin moment (I) of two proton in water、this value is related to be dipole moment (*μ*_D_) to be *μ*_D_ = 1.84 x10^-18^ esu [30]. In the case of H_2_, the electric dipole moment was calculated to be Θ_H2_ = 0.60 x10^-26^ esu [31,32], and the N_2_ is based on a triple bond but, even in this case, the electrons in the molecule are localized, and so this molecule has a quadrupole moment of Θ_N2_ = 1.5x10^-26^ esu [33–35]. These values are related to electro distribution in gas molecular. Based on the information, we calculated the excitation energy imparted by a magnetic field.

The spin quantum number (*μ*_p_) for protons in H_2_O was found from the dipole moment for water (*μ*_D_ = 1.84×10^−18^ esu). Since the relationship between the spin quantum number for protons and the dipole moment for water is *μ*_p_ = *μ*_D_^1/2^, we can calculate that *μ*_p_ = 1.36×10^−9^ esu. Since the *g* factor for protons is *g* = 2μ_p_/β_N_ = 5.586, the Bohr magneton for nuclear protons is *β*_N_ = 2μ_p_/5.586 = 4.87x10^-10^ esu. Dividing this value by the elementary charge *e* of 4.803×10^−10^ esu gives *μ*_N_ = 1.0. As a result, the spin quantum number exhibited by the two protons in a water molecule was found to be I = 1 from the dipole moment of water. Furthermore, the nuclear spin angular momentum (*I*) exhibited by the protons of water was calculated as *I* = I(I+1)^1/2^ = 1.41. In the case of H_2_, the electric quadrupole moment (Θ) has been reported to be Θ_H2_ = 0.60×10^−26^ esu. For nitrogen molecules(N_2_) that generally contain triple bonds, the quadrupole moment is treated as Θ_N2_ = 1.5×10^−26^ esu since the electrons in the molecule are localized. Based on generally accepted values for *μ*_B_ and *μ*_n_ for electrons and protons, respectively, the ratio is *μ*_B_/*μ*_N_ = 1836.4. When this value was used, the electro distribution(*μ*_e_) in a multipole molecule could be calculated from the relation of *μ*_e_ = {(2 x Θ x (*μ*_B_/*μ*_N_)^2^}^1/2^, and the magnetic moments (spin quantum number:S) for nitrogen and hydrogen were determined. For example, the spin moment exhibited by H_2_ is *μ*_*e* (H2)_ = {2x Θ_*H2*_ x (1836.4)^2^}^1/2^ = 2.92×10^−10^ esu. Since the *g* factor for electrons is *g* = 2μ_e_/β_e_ = 2.001, S _(H2)_ = 0.61 was found from the ratio of this value to the elementary charge for an electron (*e*) of 4.803×10^−10^ esu. Similarly, S_(N2)_ = 0.94 for nitrogen molecules. In this way, the nuclear spin quantum number exhibited by water and the spin quantum numbers for hydrogen and nitrogen could be found from the measured values of the dipole and quadrupole moments for gas molecules, respectively. The amount of energy (Δ*E*) excited by the magnetic field was calculated based on this basic information [29].

### Theoretical analysis of the magnetic excitation energy of dissolved gases

The ΔE values for oxygen molecules in the presence of a magnetic field can be obtained from following equation based on EPR theory.


$$\Delta E=h\nu =(\mu_{e}/S)\;H_{0}$$ (1)

This same value can also be derived from NMR theory as shown in Eq (2).


$$\Delta E=h\nu =(\mu_{N}/I)\;H_{0}$$ (2)

Here, *S* and *I* are the spin angular momentum and the nuclear spin angular momentum.

The oxygen molecule has two unpair electron in the molecule. Based on the two unpaired electrons and the electromagnetic characteristics of the molecule (e^-^), the spin magnetic moment (S) will have a value of 1. In the present work, S was determined by EPR analysis of gaseous oxygen to ensure accurate calculations. Because the water molecule (H_2_O) also has two protons (n^+^), the nuclear spin magnetic moment is I = 1. In these equations, h is Planck’s constant, ν is the resonance frequency, *μ*_e_ is the Bohr magneton and *μ*_N_ is the nuclear magnetic efficiency, with values of *μ*_e_ = - 9.27 x 10^−24^ J T^-1^ and *μ*_N_ = 1.41x 10^−26^ J T^-1^. The *I* and *S* values for hydrogen and electron were also calculated based on the relationships *I* = {I (I + 1)}^1/2^ and *S* = {S (S + 1)}^1/2^.

The excitation energy values for the two electrical polar molecules were determined using the following equations.


$$\Delta E=h\nu =(\mu_{e}/S_{H2})\;H_{0}$$ (3)

$$\Delta E=h\nu =(\mu_{e}/S_{N2})\;H_{0}$$ (4)

In addition, the spin angular momentum for a molecule having an electric dipole was obtained from the formula *S*_*H2*_ = {S_H2_(S_H2_ + 1)}^1/2^ and the spin angular momentum for an electric quadrupole was calculated as *S*_N2_ = {S_N2_(S_N2_ + 1)}^1/2^. The excitation energy (ΔE) was calculated for a magnetic field of 1.0 x 10^−4^ T.

## Results and discussion

### Magnetic properties of oxygen gas

**Fig 1:**
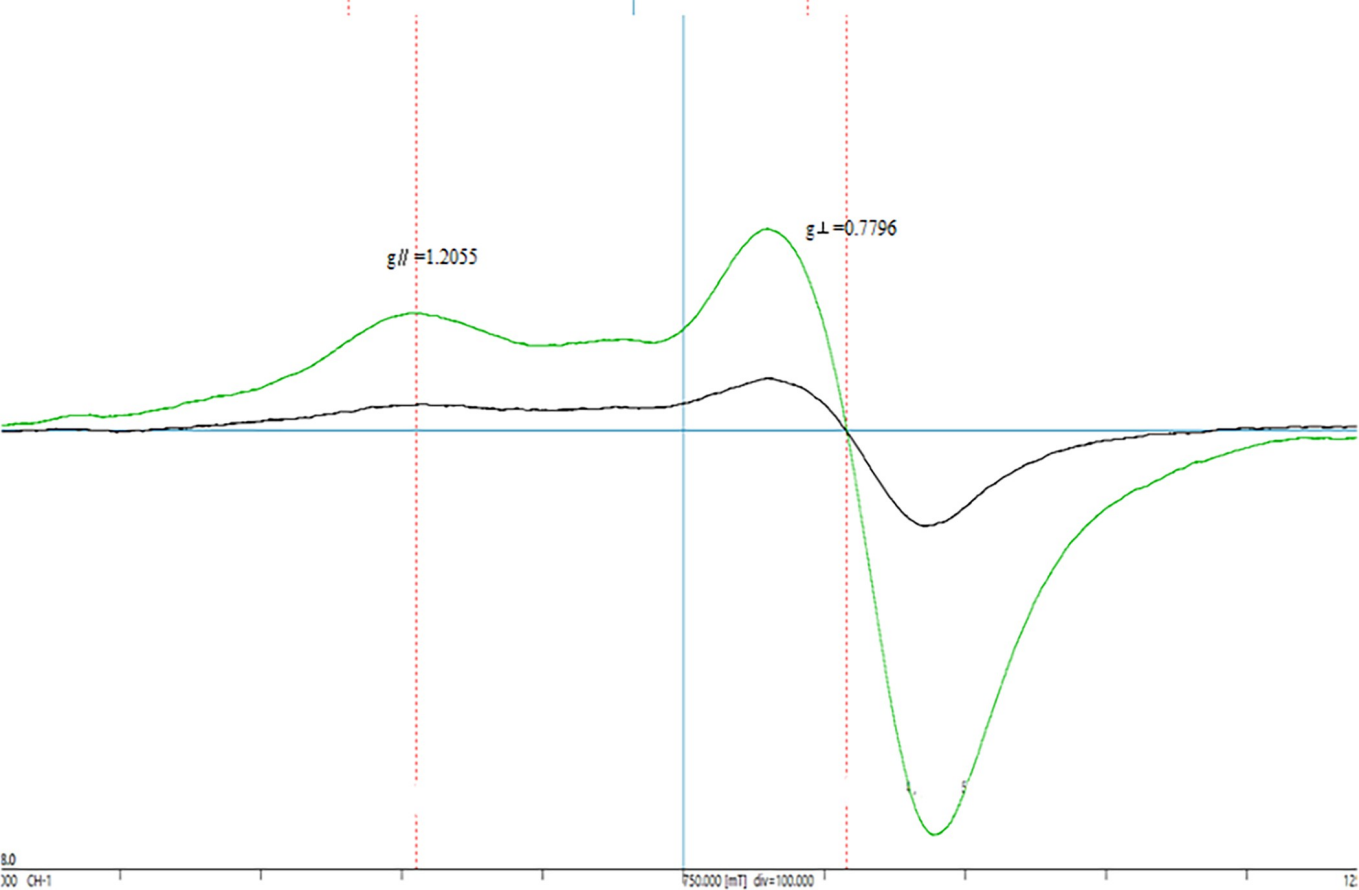
EPR spectra of pure gaseous oxygen and of air acquired under ambient conditions. The former and latter generated strong and weak peaks, respectively, for oxygen. EPR conditions: Resonance frequency 9.4566 GHz, resonance magnetic field 750 ± 500 mT, magnetic field modulation 100 kHz, modulation width 0.2 mT, amplification rate 100×, time constant 0.3 s, observation time 4 min.

Fig 1 presents EPR spectra of pure gaseous oxygen and of air, which demonstrate that gaseous oxygen is a paramagnetic molecule.

The EPR spectrum of oxygen acquired under reduced pressure (several mm Hg) exhibited nearly 280 resonance signals over a wide range of magnetic field values, due to the rotational motion of the oxygen molecules [36]. However, the concentration of pure oxygen under these conditions was approximately 40 mmol/l, and the spectrum showed a broad anisotropic line shape because of spin-spin exchange interactions between oxygen molecules, which have an ellipsoidal rather than a spherical structure. Evaluation of the EPR spectrum determined that the anisotropic g-values were g_∥_ = 0.7797 and g_⊥_ = 1.2055. In addition, the isotropic parameter was g_0_ = (2g_∥_+ g_⊥_) / 3 = 1.06. The spin quantum number for oxygen could then be determined from the g-value, and Eq (5) was used to find both the g-value and S. In the EPR equation, the spin moment S for a free radical with one unpaired electron is ½, while the g-value is approximately 2. Because oxygen has two unpaired electrons, the spin moment is S = 1, but S was calculated from the experimental values using the resonance Eq (5).


$$\Delta E=h\nu =g\beta H_{0},\;g=1/S$$ (5)

Here, ΔE is the resonance energy, h is Planck’s constant, ν is the resonance frequency, β is the Bohr magneton and H_0_ is the resonance magnetic field. The spin quantum number determined for oxygen in this manner was S = 0.94.

### Bond dissociation energies for dissolved gases

The concentrations of oxygen and hydrogen dissolved in ultrapure water were measured using the electrode method and are presented in Table 1. Note that the bulk water (nH_2_O) and nitrogen concentrations provided here were obtained from previously reported values. The temperatures in this table are the averages of two measurements.

**Table 1:** Dissolved gas concentrations at specific temperatures (n = 2).

Temperature (°C)	Cluster number of nH_2_O **)	O_2_(mol/l)	H_2_(mol/l)	N_2_ (mol/l)*)
5	19.0	0.00159	0.00093	
10	19.8	0.00153	0.00088	0.00064
15	22.0	0.00137	0.00085	
20	24.8	0.00122	0.00081	0.00052
25	27.6	0.00110	0.00078	0.00048
30	31.0	0.00098	0.00074	0.00043
35	34.9	0.00087	0.00072	
40	39.8	0.00076	0.00069	0.00037
50	42.2	0.00072	0.00063	0.00033

*^)^ Nitrogen concentrations were obtained from the Basic Handbook of Chemistry, Chemical Society of Japan [37]

**^)^ The number of water clusters was calculated from the relative concentration ratio of oxygen dissolved and bulk water. It is a value calculated assuming that the molecular bond between water and oxygen maintains a steady state at the ratio 1836.4:1 due to the Coulomb force due to electric charge.

As shown in Table 1, the concentration of the dissolved gas decreased as the temperature increased, although the values were found to become almost constant at 50°C and above. The change in the concentration of the three dissolved gases was also determined to be reversible.

**Fig 2:**
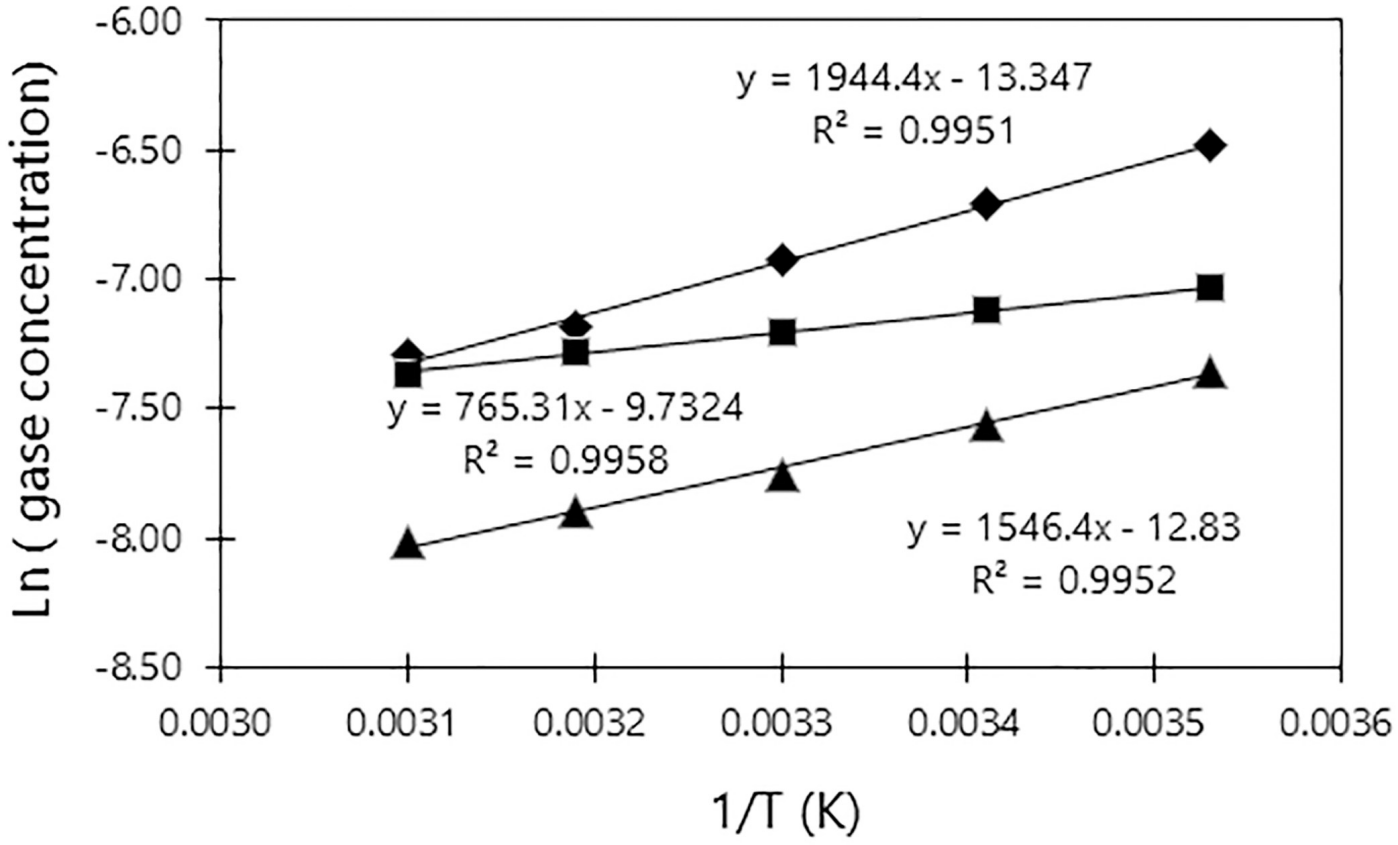
Arrhenius plots for dissolved gases in pure water. Legend: ◆: O_2_, ■: H_2_, ▲: N_2_.

The Gibbs energy changes (ΔG) for the dissolutions of these gases in water and for their dissociation could be obtained from these data, and the values for oxygen, nitrogen and hydrogen were found to be +3.84 kcal/mol (+16.1 kJ mol^-1^, +0.17 eV), +3.05 kcal mol^-1^ (+12.7 kJ mol^-1^ l, +0.13 eV) and +1.52 kcal mol^-1^ (+6.36 kJ mol^-1^ +0.016 eV), respectively. The ΔG value for oxygen was 1.26 times higher than that for nitrogen, and 2.53 times higher than that for hydrogen, indicating that oxygen molecules would be expected to be more soluble in water. On the other hand, ΔG of bulk water was -13.3 kcal mol-1 (-55.8 kJ mol-1, -0.58 eV), which was obtained from the temperature change of the dissociated oxygen concentration as shown in Table 1. As explained by electromagnetic theory, negatively charged gas molecules diffuse into positively charged water molecules. From Fig 2, it is evident that oxygen and hydrogen have the same potential energy values at 54°C, and this phenomenon may be related to the mechanism by which these two gases dissolve. Specifically, these data suggest that oxygen and hydrogen are involved in forming the intermolecular bonds known as hydrogen bonds. In contrast, the saturation of nitrogen in water occurs differently from that of oxygen or hydrogen. However, it is clear that the ΔG value for nitrogen changes with temperature in the same manner as observed for oxygen and hydrogen.

### Potential energy values (ΔE) for gases excited by the Earth’s magnetic field

Water molecules are diamagnetic and are known to adopt a cluster structure, while oxygen is paramagnetic and hydrogen and nitrogen have an electric dipole and quadrupole moment, respectively, with the latter possessing two unpaired electrons. The fact that water molecules are diamagnetic indicates that these molecules have dipoles associated with protons. As such, they will have a resonance energy based on Zeeman splitting by the Earth’s magnetic field and intramolecular charges. Using Eq (1), the resonance energy was calculated for dissolved oxygen excited by the Earth’s magnetic field, with a concentration of 1.10 mmol in water at 25°C. The Earth’s magnetic field (H_0_) in these calculations was assumed to be 1.0 x 10^−4^ T. Because the oxygen molecule has two unpaired electrons and a spin angular momentum of S = 0.94, *S* was calculated as {S (S + 1)} ^1/2^ = 1.350. The resonance energy was determined for H_0_ = 1.00 x 10^−4^ T and a water temperature of 25°C as follows.


$$\Delta E=(\mu_{e}/S)\;H_{0}=(‐9.27\;x\;10^{‐24}/1.350)\;x\;1.00\;x\;10^{‐4}=‐6.87\;x\;10^{‐28}\;J$$ (6)

This energy was then multiplied by the dissolved oxygen concentration at 25°C (Table 1) and by Avogadro’s number as shown below.


$$\Delta Ep\;(O_{2})=‐6.87\;x\;10^{‐28}\;x\;1.10\;x\;10^{‐3}\;x\;6.02\;x\;10^{23}=‐4.55\;x\;10^{‐7}\;J\;mol^{‐1}$$ (7)

This value is the magnetic energy for dissolved paramagnetic oxygen molecules in the Earth’s magnetic field.

Similarly, the electric dipole moment of dissolved hydrogen was calculated as S_H2_ = 0.61　and *S*_H2_ = {S_H2_ (S_H2_+1)}^1/2^ = 0.99.


$$\Delta Ep\;(H_{2})=(\mu_{e}/S_{E})\;H_{0}=(‐9.27\;x\;10^{‐24}/0.99)\;x\;1.00\;x\;10^{‐4}=‐9.36\;x\;10^{‐28}\;J$$ (8)

The above was based on Eq (3) and was multiplied by the concentration of dissolved hydrogen at 25°C (0.78mmol) and Avogadro’s number as below.


$$\Delta Ep\;(H_{2})=‐9.36\;x\;10^{‐28}\;x\;0.78\;x\;10^{‐3}\;x\;6.02\;x\;10^{23}=‐4.40\;x\;10^{‐7}\;J\;mol^{‐1}$$ (9)

Furthermore, the quadrupole moment of nitrogen when dissolved was calculated as S_Q_ = 0.94 and *S*_N2_ = {S_N2_(S_N2_+1)}^1/2^ = 1.35, giving the following.


$$\Delta E\;(N_{2})=(\mu e/S_{N2})\;H_{0}=(‐9.27\;x\;10^{‐24}/1.35)\;x\;1.00\;x\;10^{‐4}=‐6.87\;x\;10^{‐27}\;J$$ (10)

This leads to Eq (11), based on a dissolved nitrogen concentration of 0.48 mmol and again multiplying by Avogadro’s number.


$$\Delta Ep\;(N_{2})=‐6.87\;x\;10^{‐27}\;x\;0.48\;x\;10^{‐3}\;x\;6.02\;x\;10^{23}=‐1.98\;x\;10^{‐7}\;J\;mol^{‐1}$$ (11)

The results from Eqs (7) and (8) were compared to those obtained by substituting Eq (9) into Eq (11). This showed about same between the magnetic energy values (ΔEp) for dissolved oxygen and dissolved hydrogen, and that the excitation energy (ΔEp) for dissolved nitrogen was 0.44 times that for oxygen.

The magnetic energy calculations for diamagnetic water molecules were performed using Eq (2). The nuclear spin quantum number for two protons in a water molecule (nH_2_O) is I = 1 and, nuclear spin angular momenta was *I =* {I(I+1)}^1/2^ = 1.41.


$$\Delta E=(\mu_{N}/I)\;H_{0}=(+1.41\;x\;10^{‐26}/1.41)\;x\;1.00\;x\;10^{‐4}=+1.00\;x\;10^{‐30}\;J$$ (12)

Using the value from Eq (12) and assuming a cluster number of 28 the water concentration at 25°C is 2.04 mol/l, and this value could be employed to calculate the NMR energy as follows.


$$\Delta Ep\;(nH_{2}O)=+1.00\;x\;10^{‐30}\;x\;2.01\;x\;6.02\;x\;10^{23}=+1.21\;x\;10^{‐6}\;J\;mol^{‐1}$$ (13)

Using the method described above and the concentrations of dissolved gases in Table 1, the excitation energy (ΔE) values associated with a magnetic field were obtained, as presented in Table 2.

**Table 2:** Excitation energies provided by the Earth’s magnetic field at specific temperatures.

Temp. (°C)	nH2O(x10-7) JT-1	O2 (x10-7) JT-1	H2 (x10-7) JT-1	N2 (x10-7) JT-1
5	17.57	6.57	52.36	
10	16.89	6.31	49.54	26.43
15	15.18	5.67	47.86	
20	13.47	5.03	45.60	21.48
25	12.12	4.53	43.91	19.82
30	10.78	4.03	41.66	17.76
35	9.58	3.58	40.54	
40	8.39	3.14	38.85	15.28
50	7.93	2.96	35.47	13.63

As is shown in Table 2, it is evident that the excitation energies of the oxygen molecules are lower than the value for bulk water at temperatures between 10 and 40°C, so that oxygen are retained by the water molecules and dissolve. Furthermore, the excitation energy of nitrogen is about four times that of oxygen, suggesting that the ratio of both dissolved in water is related to the partial pressure of gas concentration in the atmosphere. It can be said that the excitation energy of hydrogen is higher than that of water and does not dissolve stably. In addition, it can be investigated the correlation between the excitation energy of water molecules and the dissolution of the gases. The Gibbs energy (ΔG) values calculated from the concentration-induced changes and magnetic moment of dissolved paramagnetic gas molecules and bulk water are provided in Table 3.

**Table 3:** Gibbs energy (ΔG) and magnetic moments for dissolved gases.

Molecule	ΔG (kJ mol^-1^)	Magnetic moments (S and I) (JT^-1^)
O_2_	+16.1	-0.94
N_2_	+12.7	-0.94
H_2_	+6.36	-0.60
nH_2_O	-55.4	+1.00

In Table 3, the nuclear magnetic moment (I = +1.00) of bulk water (nH_2_O) is the product value of the nuclear spin quantum number and the nuclear magnetic efficiency (μ_n_), and the magnetic moment (S = -0.94) of oxygen is the electron spin quantum number and Bohr magneton (μ_B_). Hydrogen and nitrogen, which are non-polar molecules and quadrupole molecules, were calculated as having the same electron spin characteristics as oxygen.

### Relationship between the magnetic moment and magnetically excitation energy

The electromagnetic effect generates a repulsive force between charges of the same polarity, while there will be an attractive force between opposite charges. The data in Table 2 and the diamagnetic energy for water molecule clusters in the presence of the Earth’s magnetic field, as well as the paramagnetic energy values for oxygen and hydrogen, were found to be in agreement. These results for dipolar water molecules and for paramagnetic oxygen along with nitrogen and hydrogen, which are quadrupolar and nonpolar molecules, respectively, indicate the possibility of magnetic coupling. Thus, the effect of the planet’s geomagnetic field on binding between molecules was confirmed.

**Fig 3:**
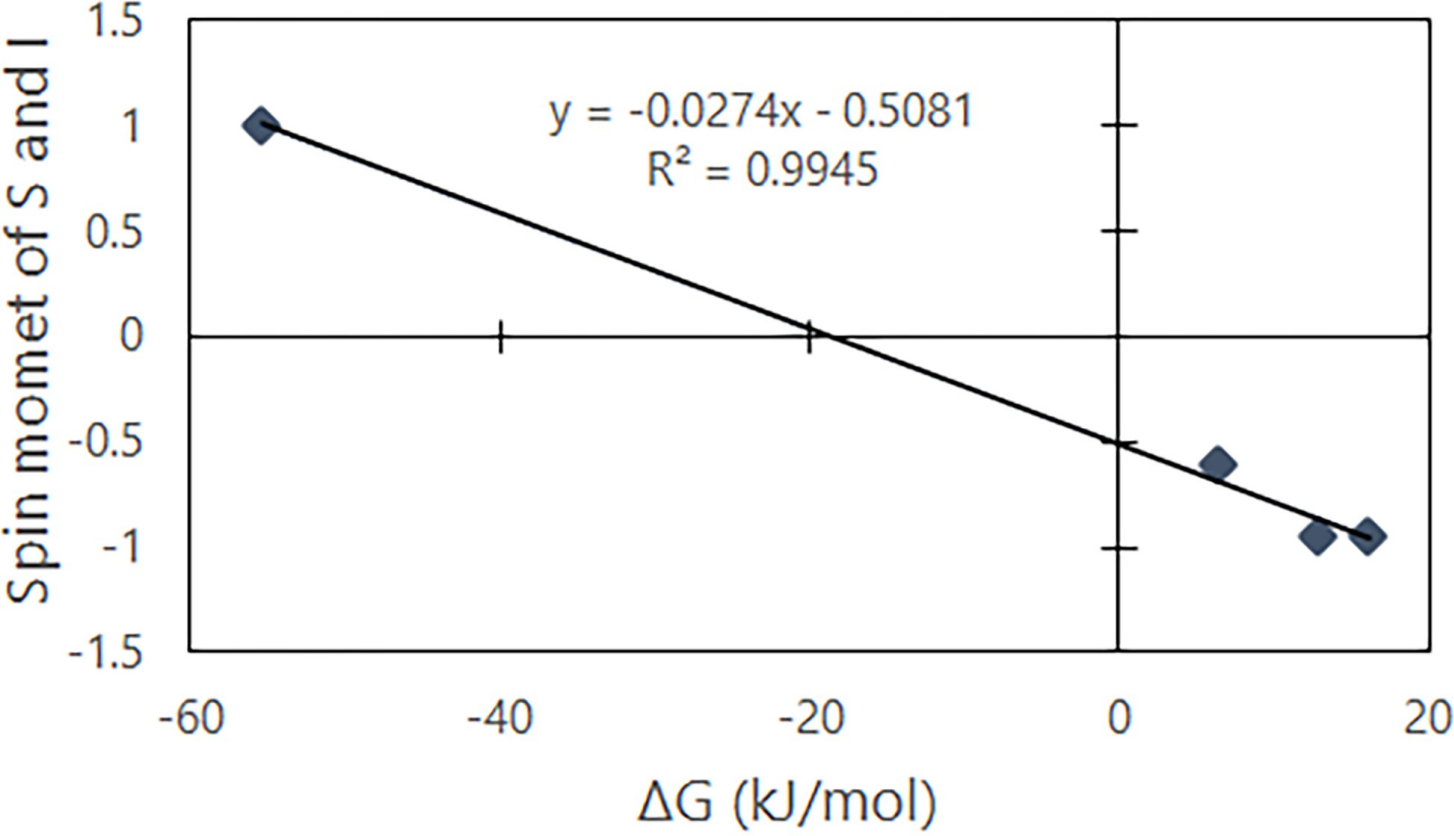
Relationship between the magnetic moments of gas molecules and gibs energy.

The dissolution of gas molecules in water can be explained by employing the theory of electromagnetism. Electromagnetism generates a repulsive force between charges having the same polarity while charges with opposite polarities will be attracted to one another. The magnitude of the force is proportional to the product of the charges and inversely proportional to the square of the distance. The fundamental principles of magnetic coupling can be understood by examining the relationship between the thermodynamic behavior and electromagnetic properties of gas molecules dissolved in water. In the model used for the present calculations, water molecules are diamagnetic and have a cluster structure while oxygen is paramagnetic with two unpaired electrons in the molecule. Hydrogen and nitrogen are treated as quadrupole molecules that exhibit electrical polarity, and water molecules have a dipole moment based on the presence of two protons. As a result of the Earth’s magnetic field, these molecules undergo Zeeman splitting along with the effect of intramolecular charges, and so will show a magnetic resonance energy. The energy values were determined using a magnetic field strength of 1.00 x 10^−4^ T, taking into account the magnetic moments for oxygen, hydrogen and nitrogen together with basic quantum chemistry calculations and magnetic resonance theory. As shown in Fig 3, the Gibbs energy values for gaseous oxygen, hydrogen and nitrogen dissolved in water showed significant correlation (R^2^ = 0.9946) with the molecule-specific magnetic moments. This result suggests that the difference between the positive electric properties of water molecules and the negative electric properties of oxygen, hydrogen and nitrogen are involved in intermolecular bonding.

## Conclusions

The dissolution of gas molecules in water was determined to involve an exchange of energy in response to excitation by a magnetic field. Variations in the solubility of several gases with temperature were also reproduced by quantum chemistry calculations, using a magnetic field strength of 1.00 x 10^−4^ T, which is equivalent to that of the Earth magnetic field. The bond between water and gas molecules was an electric charge-dependent Coulomb bond. And also, The hydrogen bonds and van der Waals forces in water were determined to reversibly generate cluster structures as a consequence of the effects of both the Earth’s magnetic field and temperature. The results of this work provide new information concerning intermolecular interactions that have implications in a wide range of research within the field of chemistry.
